# Reflection Leading to Action on Diversity, Equity, and Inclusion at the Virginia GWEP

**DOI:** 10.1093/geroni/igab046.341

**Published:** 2021-12-17

**Authors:** Chuck Alexander, Patricia Slattum, Ishan Williams, Leland Waters

**Affiliations:** 1 Virginia Commonwealth University, Richmond, Virginia, United States; 2 Department of Pharmaceutics, Richmond, Virginia, United States; 3 University of Virginia, Charlottesville, Virginia, United States

## Abstract

Last year’s Black Lives Matter protests inspired the Virginia Geriatric Education Center (VGEC) GWEP’s plenary to engage in reflection and discussion on diversity, equity, and inclusion (DEI) in our work together. During each bi-monthly meeting, we dedicate time to generate ideas to improve our programming, how we work together, and how we partner and recruit for our programs. Champions for DEI on our plenary led an effort to develop a DEI newsletter clarifying DEI concepts and introducing resources thematically related to the monthly VGEC faculty development program curriculum. By incorporating these resources into our monthly curriculum, facilitators have a new access point to incorporate content on health equity and policy into our curriculum. The intentional focus on DEI is opening the door to deeper reflection and conversation with a goal of improving all our programming, cultivating a new social awareness, and bringing new voices and perspectives to the table.

